# Current Status of Alginate in Drug Delivery

**DOI:** 10.1155/2020/8886095

**Published:** 2020-08-06

**Authors:** Dewi Melani Hariyadi, Nazrul Islam

**Affiliations:** ^1^Pharmaceutics Department, Faculty of Pharmacy, Airlangga University, Nanizar Zaman Joenoes Building, Jl. Mulyorejo Campus C, Surabaya 60115, Indonesia; ^2^School of Clinical Sciences, Queensland University of Technology, Brisbane, Australia; ^3^Institute of Health and Biomedical Innovation (IHBI), Queensland University of Technology, Brisbane, QLD, Australia

## Abstract

Alginate is one of the natural polymers that are often used in drug- and protein-delivery systems. The use of alginate can provide several advantages including ease of preparation, biocompatibility, biodegradability, and nontoxicity. It can be applied to various routes of drug administration including targeted or localized drug-delivery systems. The development of alginates as a selected polymer in various delivery systems can be adjusted depending on the challenges that must be overcome by drug or proteins or the system itself. The increased effectiveness and safety of sodium alginate in the drug- or protein-delivery system are evidenced by changing the physicochemical characteristics of the drug or proteins. In this review, various routes of alginate-based drug or protein delivery, the effectivity of alginate in the stem cells, and cell encapsulation have been discussed. The recent advances in the in vivo alginate-based drug-delivery systems as well as their toxicities have also been reviewed.

## 1. Introduction

### 1.1. Chemistry and Physicochemical Properties of Alginate

**Figure 1:**
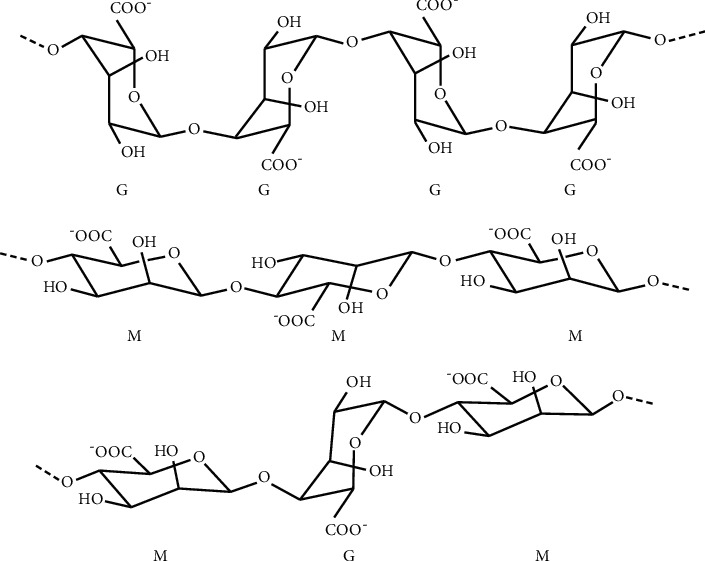
Chemical structures of G-block, M-block, and alternating block in alginate [1].

Alginate is a polysaccharide extracted from brown seaweeds, including *Laminaria hyperborea*, *Laminaria digitata*, *Laminaria japonica*, *Ascophyllum nodosum*, and *Macrocystis pyrifera* [1, 2]. It is composed by a sequence of two (1Ñ4)-linked *α*-L-guluronate (G) and *β*-D-mannuronate (M) monomers. The proportion of M and G blocks may vary with the type of seaweed from where it is extracted (Figure 1). For example, alginate extracted from *Laminaria digitata* and *Ascophyllum nodosum* has been shown to have M/G ratios of 1.16 and 1.82, respectively. Alginate is a biocompatible polymer with very low toxicity [3]. These are the main advantages that make alginate one of the biopolymers with the widest biomedical applicability [4, 5]. One of the most common applications of alginate is their use as an excipient in drug-delivery systems, namely, acting as a stabilizer agent in various pharmaceutical formulations [6, 7].

Alginate has carboxyl groups which are charged at pH values higher than 3-4, making alginate soluble at neutral and alkaline conditions to promote the widespread use of alginates. For some drugs which require greater protection with preferential absorption in the intestinal tract or other conditions such as modified drug release, alginate is a preferable polymer. Thus, solubility and pH sensitivity make alginate a good biomaterial for drug-delivery systems [8]. Sodium alginate is the type of alginate mainly used in the pharmaceutical industry and may be used for the purpose of extending the drug release. Using sodium alginate with different chemical features and degree of viscosities, the slow release of ibuprofen from press-coated tablets was reported [8]. In acidic environments, alginate carboxyl groups are protonated, thereby limiting drug release. Alginate has the ability to crosslink with Ca^2+^ ions through an ionotropic gelation process, usually above pH 6. Ba^2+^ or Zn^2+^ ions are also used as crosslinkers [9–11].

Alginate hydrogels are applied in wound healing treatments through the construction of wound dressings [12–15]. Several studies showed that the bioavailability of drugs encapsulated in alginate hydrogels is greater than that of the free drug applied directly at the lesion site, thus increasing the efficacy of healing. Alginate hydrogels are also used widely in tissue regeneration treatments and cell encapsulation [16–22]. Alginate may be used in the construction of capsules for cell encapsulation often associated with cytotherapy treatments or simply the creation of cellular microcultures in more complex systems. A new approach to the construction of alginate-based capsules for the incorporation of different types of cells has been demonstrated [23]. Cells were encapsulated in alginate liquefied particles, followed by coating it with chitosan and alginate. Poly(lactic acid) microparticles along with the cells were coencapsulated to protect cell survival with high viability of the encapsulated cells. Hydrogels obtained from alginate nowadays present some advantages of being appropriate materials to be used in tissue engineering and regenerative medicine applications [23–31].

Some important uses of alginates in nanomedicines in the forms of dendrimers, nanocrystals, emulsions, liposomes, solid lipid nanoparticles, micelles, and polymeric nanoparticles have provided advantages over conventional medicines including efficacy, safety, physicochemical properties, and pharmacokinetic/pharmacodynamic profiles [32].

### 1.2. Crosslinker for Alginate Micro/Nanoparticles to Encapsulate Drugs

Typical shapes of alginate are processing through several different techniques, including emulsion, multiple-phase emulsion, and cation crosslinked encapsulation (Ca^2+^, Ba^2+^, or Cu^2+^) [33–37]. The ability of alginate to create complexes with other biomaterials by electrostatic interactions, chemical modification, or crosslinking can be exploited for building hybrid and more versatile DDSs. Capsules constructed from chitosan/alginate-PEG complexes are reliable models for encapsulating proteins, such as albumin, one of the most common model proteins used in controlled release studies [38–43]. This approach can promote higher control release of drugs, proteins, and other biomolecules.

#### 1.2.1. Effect of Different Classes of Crosslinkers on Alginate Polyelectrolyte Nanoparticle

Mirtic et al. [10] investigated the preparation of alginate nanoparticles using complexation of different classes of crosslinkers (divalent cations, polycations, and positively charged surfactants) and found that alginate nanoparticles were formed across a limited range of molar ratios that were specific for each crosslinker and had different size and stability. Additionally, the ionic strengths of the media influenced the characteristics and stabilities of the polyelectrolyte nanoparticles.

#### 1.2.2. Effect of Divalent Cation on Morphology and Drug-Delivery Efficiency

A study by Deepika et al. [44] was about the formation of levofloxacin in chitosan-alginate hybrid gel for controlled release and effect of divalent alkaline ions (Mg^2+^, Ca^2+^, Sr^2+^, and Ba^2+^) on encapsulation efficiency and drug release kinetics from chitosan-alginate nanostructure was investigated. The particle size increases and encapsulation efficiency decreases with the size of the divalent ions. Spherical shaped particles were formed by Mg^2+^ and Ca^2+^, whereas Sr^2+^ and Ba^2+^ produced nonspherical particles. Transformation of microspheres is shown by SEM as truncated tetrahedron by Sr^2+^ and clear rod shape by Ba^2+^ was identified. This suggested that metal ions have a significant influence on the morphology, drug encapsulation, and release profile of the chitosan-alginate hybrid polymer nanoparticles.

#### 1.2.3. Effect of Zinc-Ion Complex with Alginates

Kotagale et al. [45] complexed alginates with zinc metal ion to improve beads' physicochemical and biological properties for controlling the drug release. They found that the atenolol-zinc polymeric beads exhibited pulsed release with increased half-life. Moreover, no significant differences in in vitro and in vivo atenolol release behavior among the *N*,O-dimethyl, *N*-methyl, or *N*-benzyl hydroxylamine derivatives of sodium alginate were observed.

#### 1.2.4. Effect of Ferric Ion Crosslinker on Alginates

Microspheres of acrylamide- (AAm-) grafted poly(vinyl alcohol) (PVA)/sodium alginate (NaAlg) were prepared by crosslinking with FeCl_3_ and 5-fluorouracil (5-FU) [46]. Microspheres were characterized by particle diameter, equilibrium swelling values and morphology, elemental analysis, and release profiles. This group studied the effects of PVA-g-PAAm/NaAlg ratio, drug/polymer ratio, crosslinker concentration, and exposure time to FeCl_3_ on the release of 5-FU. The highest 5-FU release was found to be as 99.57% after 6 h for PVA-g-PAAm/NaAlg and release kinetics was described by Fickian and non-Fickian approaches.

### 1.3. Purposes of Encapsulation of Drugs Using Alginates

Alginate can also undergo complexation with natural polymers, like chitosan, to enhance the absorption and cargo protection in oral delivery, for example, for the administration of insulin [47, 48]. Alginate was also combined with pectin polymer which has a similar mechanism. This research also showed successfully encapsulated drugs [49–52]. Alginate-based drugs encapsulated into nanoparticles/microparticles with various purposes are presented in Table 1.

### 1.4. Use of Alginates in the Pharmaceutical Industry

Many application areas of sodium alginate-based drug-delivery systems, and these systems can be formulated as gels, matrices, membranes, nanospheres, microspheres, and others [2, 81]. Researchers are exploring possible applications of alginates as a coating material and preparation of controlled release drug-delivery systems.

#### 1.4.1. Alginate for Protein Delivery and Cell Encapsulation

Alginate microparticles as a carrier for protein delivery prepared by spray-drying processes have been studied for their application in nasal and pulmonary drug delivery [85–87] prepared inhalable alginate particles (of an average diameter 3.23 ± 0.25 *μ*m) with a high encapsulation efficiency of 97% with the preserved structure and bioactivity of BSA. The alginate particles released approximately 20% of the loaded BSA over 24 h and then a slow release occurred, reaching a cumulative release of only 35% after 180 h. Möbus et al. [88] prepared Zn^2+^-crosslinked alginate microparticles containing the model protein BSA via a simple one-step spray-drying process to produce microparticles of 2–4 *µ*m size. They found BSA release into the simulated lung fluid increased with an increasing content of protein in the alginate microparticles. Alginate hydrogels have also been studied for oral delivery of proteins [89, 90]. Hariyadi et al. [91] prepared alginate microspheres containing lysozyme and insulin resulting in 30 to 60 *μ*m in size with high protein loadings. Moreover, it was found to retain 75% activity using the ARCHITECT® assay and exhibit at least 80% bioactivity using the *Micrococcus lysodeikticus* assay. Another study using BSA demonstrated that the BSA release from the hydrated microparticles reached less than 7% in the simulated gastric fluid over 2 h, whereas 90% of the protein load was gradually released in the simulated intestinal fluid over 10 h. Another cell viability study was also conducted by Morachis et al. [92]; Severino et al. [93]; Joddar et al. [94]; Ciriza et al. [95]; Yoncheva et al. [96]; and Gurruchaga [18]. Applications of alginates for protein delivery and cell encapsulation are presented in Tables 2 and 3.

#### 1.4.2. Alginate Particles with Ovalbumin (OVA)

*(1) Peptide as a Carrier and Adjuvant*. Ovalbumin (OVA) peptide 323–339 encapsulated in alginate has been reported to be involved in immune response as carrier and adjuvant for the immune therapy of cancer [53]. A tumor model was established in C57BL/6J mice via subcutaneous injection of 3 × 105 B16-OVA tumor cells. Alginate/OVA peptide inhibited tumor progression more effectively than using the peptide alone. The viability and uptake study illustrated that this particle is safe and nontoxic. Furthermore, alginate particles can promote the activation of surface markers on macrophages. ELISA assay showed that the particles with peptide can promote the secretion of inflammatory and effector cytokines from macrophages.

#### 1.4.3. Liposomal Alginate for Bupivacaine Delivery and MSC Function

Mesenchymal stromal cell (MSC) therapies have become potential treatment options for multiple ailments and traumatic injuries. Davis et al. [103] developed and characterized a sustained release delivery formulation comprised of alginate-encapsulated liposomal bupivacaine to evaluate the effect of this formulation on the secretion of three key MSC regulatory molecules, interleukin 6 (IL-6), prostaglandin E2 (PGE2), and transforming growth factor-beta 1 (TGF-*β*1). Bupivacaine release profile analyses indicated that the mode of drug delivery controlled the liposomal-alginate (LA) concentration over time and pathway analysis identified several shared and cytokine-specific molecular mediators for IL-6, PGE2, and TGF-*β*1. These studies support the potential utility of LA for anti-inflammatory cell therapy coadministration.

#### 1.4.4. Curcumin-Alginate-Based Composite Sponges

Alginate-based composite sponges were developed as carriers to prolong the gastric retention time and controlled release of curcumin-loaded self-microemulsifying drug-delivery systems (Cur-SMEDDS) [104]. Researchers used adsorbent (colloidal silicon dioxide) and additional polymers such as sodium carboxymethyl cellulose (SCMC) and hydroxypropyl methylcellulose (HPMC) to form composite sponges. The formulation exhibited a droplet size of approximately 30 nm and provided a sustained release.

## 2. Application of Alginates in Context of the Routes of Drug Administration

Alginates have been extensively investigated for delivering drugs via oral, parenteral, pulmonary, and transdermal routes (Table 4). Using alginate as a single polymer or the combined polymer, controlled or sustained release delivery of quercetin, isoniazid, rifampicin, ciprofloxacin, bovine insulin, and lentivectors has been investigated. All formulations showed increased entrapment efficiency of drugs, increased dissolution and bioavailability, and reduced degradation of drugs [105–107, 109–112, 130–132]. Some chemotherapeutic agents encapsulated in alginate polymer showed enhanced penetration in the target cells. Antigen-encapsulated alginate showed enhanced immune response [8, 115, 116, 133, 134]. Alginates have been also widely investigated for pulmonary drug delivery [99, 117, 119–128]. Alipour et al. developed paclitaxel-alginate microparticles which increased the site-specific efficacy of drugs with reduced toxicity [117]. Using alginate and PLGA polymers, Abdelaziz et al. studied inhalable particulate delivery of cisplatin and doxorubicin for lung cancer therapy [120]. The alginate-based BSA and BCG vaccines have been used to study the efficacy of smaller inhalable vaccines, which provided better protection and more immunogenic effect [99, 124, 125]. Applications of alginate in transdermal delivery for wound dressing or wound healing were shown to be effective to produce a high porosity and sustained release and able to inhibit preinfection [126–128, 135].

### 2.1. Alginate-Based Hybrid Aerogel Microparticles for Mucosal Drug Delivery

Some polysaccharides (e.g., alginate, chitosan, and pectin) have been applied as biopolymer aerogels to have mucoadhesive properties for mucosal drug delivery [136] Alginate-based hybrid aerogels of microparticles (<50 *μ*m) were produced. Low methoxyl pectin and *κ*-carrageenan were also cogelled with alginate and further dried with supercritical CO_2_ (sc-CO_2_). Spherical mesoporous aerogel microparticles were obtained for alginate, hybrid alginate/pectin, and alginate/*κ*-carrageenan aerogels, presenting high specific surface area and mucoadhesive properties. The microparticles were loaded with ketoprofen and quercetin. Release of both drugs from alginate/*κ*-carrageenan aerogel was slightly faster compared to alginate/pectin indicating that alginate-based aerogel microparticles are potential for mucosal drug-delivery applications.

### 2.2. Alginates for Ocular Drug Delivery

To develop potential ocular drug delivery, mucoadhesive microspheres is one of the best approaches to prolong the drug residence inside the cul-de-sac, consequently increasing the bioavailability. Thus, some researchers worked to overcome the limitations of ocular drug delivery [137–139]. The chitosan-sodium alginate microspheres or other polymers encapsulating of ocular drugs have been investigated widely. Sodium alginate microspheres prepared were in particle size range suitable for ocular purpose and were able to improve the therapeutic efficacy.

### 2.3. Alginates for Stem Cell Purposes

Alginates as polymer have been used for stem cell studies. For example, Leslie et al. studied the controlled release of rat adipose-derived stem cells from alginate microbead [140]. Maia et al. formed hydrogel depots for local codelivery of osteoinductive peptides and mesenchymal stem cells [141]. Another study used cartilage cells in a combination of alginate and hyaluronate hydrogels for cartilage regeneration [37, 84, 142, 143]. Ulker and Erkey studied spermatogonial stem cells and evaluated alginate hydrogel cytotoxicity on three-dimensional culture [144].

## 3. Various Techniques to Produce Alginate Micro/Nanoparticles for Drug Delivery

Over the years, various methods have been developed to fabricate drug-delivery particles of bioactive substances. Using superhydrophobic surfaces, it is possible to produce polymer particles suitable as DDSs. This method allowed loading drugs into spherical structures with an encapsulation efficiency close to 100% [145, 146]. Goncalves et al. [136] developed alginate microparticles which were shown to have perfluorocarbon breakthrough capacity when subjected to vibration by ultrasound waves. Results showed a disruption of these microparticles after 15 min of exposure, suggesting that such structures are promising DDSs controlled externally by acoustic stimuli.

Another strategy to synthesize particles relies on complexation, based on the electrostatic interactions between alginate at neutral and alkaline pH values, bioactive agents, and other kinds of naturally occurring polymers, such as the polycation chitosan [147–149].

### 3.1. Preparation Techniques for Production of Alginate Nanoparticles

#### 3.1.1. Oligopeptide-Side Chained Alginate via the Amidation Method

A melittin-targeting drug carrier was successfully synthesized by the grafting of sodium alginate to an oligopeptide via an amidation method at different oligopeptide: alginate unit molar ratios [150]. The average sizes of the oligopeptide-alginate nanoparticles formed decreased with increasing oligopeptide contents, indicating intramolecular interactions between oligopeptide-side chains. The results confirm that the derivation of an oligopeptide-side chain in alginate offers a specific binding site for melittin and effectively works in cancer chemotherapy.

#### 3.1.2. Chitosan/Alginate Nanoparticles by Emulsification and Ionotropic Gelification

Curcumin-diglutaric acid (CG) is a prodrug of curcumin encapsulated into chitosan/alginate polysaccharide-based nanoparticles [151]. CG-loaded chitosan/alginate nanoparticles were prepared by o/w emulsification and ionotropic gelification, with the conditions optimized using response surface methodology. The CG-loaded chitosan/alginate nanoparticles showed better stability compared to a CG dispersion in water. The nanoparticles showed slow cumulative release and the release pattern was mainly controlled by Fickian diffusion and erosion of polymer materials. CG-loaded chitosan/alginate nanoparticles showed higher in vitro cellular uptake in human epithelial colorectal adenocarcinoma (Caco-2 cells) and better anticancer activity against Caco-2, human hepatocellular carcinoma (HepG2), and human breast cancer (MDA-MB-231) cells.

#### 3.1.3. Alginate/Chitosan Nanoparticles for Controlled Release of Vitamin B2

Work by Azevedo et al. [152] encapsulating vitamin B2 with alginate/chitosan nanoparticles using ionotropic polyelectrolyte pregelation was conducted. Alginate/chitosan nanoparticles were 104.0 ± 67.2 nm, PDI of 0.319 ± 0.068, encapsulation efficiency, and loading capacity values of 55.9 ± 5.6% and 2.2 ± 0.6%, respectively. Sizes and PDI during 5 months showed that vitamin B2-loaded nanoparticles were stable.

#### 3.1.4. Nutraceutical Nanodelivery System

Alginate nano/microspheres were produced by emulsification/internal gelation of sodium alginate within vegetable oils containing surfactant, followed by CaCl_2_ addition resulting in hardened particles [153]. Size of nanoparticles decreased at higher oil and surfactant contents, higher molarity of CaCl_2_, and lower alginate concentrations. Moreover, encapsulation efficiency was inversely proportional to the size of nanoparticles.

#### 3.1.5. Alginate/Chitosan Formulations for Ciprofloxacin-Controlled Delivery

Kyziol et al. loaded ciprofloxacin in alginate beads with an emulsification technique in combination with an internal gelation method [154]. Hydrodynamic diameter and zeta potential showed of 160 nm and −32 mV in the case of AL_CP and ca. 240 nm and ca. +14 mV in the case of AL_CP_CS, respectively. They found that alginate beads with encapsulated ciprofloxacin covered with chitosan were effective oral delivery system since limited ciprofloxacin was release in gastric.

Various techniques which have been used to produce alginate nanoparticles are presented in Table 5.

### 3.2. Preparation Techniques for Production of Alginate Microparticles

Some techniques were used to produce alginate microparticles. Production is by conventional emulsification using sodium alginate single or combination polymer with chitosan to encapsulate a variety of drugs including glucose oxidase [167], paclitaxel [168], cocoa extract [169], and diclofenac sodium [170] or double emulsification techniques [171].

Another method is internal gelation technique, which by using sodium alginate polymer to entrap drug of doxorubicin was done by Giovagnoli et al. [35], diclofenac by Ahmed et al. [172], L-*α*-phosphatidylcholine by Semmling et al. [173], and sulfasalazine by Tavakol et al. [174]. Extrusion dripping method was also used to optimize sphericity of particles and shape deformation [175].

The more recent technique to produce microparticles was an impinging aerosol technique to successfully encapsulate propranolol HCl by Hariyadi et al. [89] and high-voltage electrostatic bead generator for BSA-alginate microparticles by Ørning et al. [176]. Mishra et al. [177] used gas blowing technique to contain verapamil HCl resulting in faster/burst drug release; however, importantly a strong mechanical strength and drug integrity were maintained in hydrogel polymeric network.

## 4. Mechanism of Drug Release from Alginate Nano/Microparticles

Some researchers focused on investigating release behavior of polymer in nanoparticles and microparticles by modified polymers which are used to form hydrogels or other ways such as producing smart polymers consisting of copolymerized agents as additional polymer, change the pH of the encapsulation process, temperature changes, and others [178–184]. James et al. designed smart polymers in order to achieve mechanism of release of swelling, contraction, and disintegration mechanism, although these additional agents must be programmable to show depot mechanism for sustained release, for example, the formation of complex from chitosan and glycerophosphate [179]. There are different mechanisms of release of a bioactive agent from the carrier, such as through variations of temperature and pH and the use of biodegradable materials or enzymatic degradation, among other chemical and physical stimuli-responsive methods [42, 185–189]. Hadijev et al. [180] studied hydrogels which mostly applied drug diffusion as a release mechanism; however, this can be changed with the properties to broadly change the solute diffusion coefficient as the gel system swells. According to Gao et al. [183], mechanism of release of hydrogels can be modified to have more steady release behavior by adding some copolymer which is able to interact and may change the chemical structure, morphology, and rheology characteristics, thus affecting release behavior and mechanism.

## 5. Toxicity and In Vivo Study

### 5.1. Toxicity

Alginate nanoparticles and microparticles were considered safe, although some studies about safety and toxicity were widely conducted. For example, Spadari et al. [120] investigated alginate nanoparticles as a nontoxic delivery system for miltefosine (MFS) in the treatment of candidiasis and cryptococcosis. Alginate nanoparticles were produced using the external emulsification/gelation method and toxicity on red blood cells and *Galleria mellonella* larvae were assessed. MFS in alginate nanoparticles presented no hemolytic effect and no toxicity in *G. mellonella* larvae. These results showed the potential and nontoxic use of alginate-based drug-delivery systems as carriers to control the fungal infection in the in vivo model of *G. mellonella*.

### 5.2. In Vivo Study for Alginate Nano/Microparticles

In vivo study is usually not directly related to the in vitro achievement. Here are some potential in vivo studies for alginate nanoparticles and microparticles. Wang et al. demonstrated that BaSO_4_/alginate microspheres possessed excellent visibility under X-ray and histopathology analysis for transcatheter arterial embolization (TAE) therapy. In vivo study verified that the embolic efficacy of microspheres was similar to that of commercially available alginate microsphere embolic agents [14]. For colon study, Patole and Pandit entrapped mesalamine in variety of polymers including alginate, HPMC, and Eudragit FS-30D and found histopathologically no signs of ulceration or bleeding of the released microspheres [190]. Other in vivo studies including anti-inflammatory, mucoadhesion test, and histopathological were conducted by researchers [191–195].

For vaccine delivery, research using chitosan, trimethyl chitosan (TMC), and alginate was conducted by Mosafer et al. using inactivated PR8 influenza virus for mucosal vaccine delivery. PR8-chitosan formulation elicited higher IgG2a and IgG1 antibody titers compared with PR8-TMC. Alginate coating significantly decreased the antibody titers and less immune response was induced [121].

In vivo study for the transdermal application was done by Hariyadi et al. [196]. They showed the effectiveness of glutathione-alginate microspheres in decreasing matrix metalloproteinase-1 (MMP-1) expression in the dermis tissue of mice.

Natural products have been investigated by researchers in vivo. Alginate polymer-encapsulated black seed oil for intestine-targeted drug delivery has been studied by Azad et al. (2020) in the forms of gastrointestinal distribution study [197]. They found uniform distribution of beads after oral administration in rats.

Beside in vivo investigation, Thai et al. indicated low toxicity of lovastatin-alginate and chitosan nanoparticles in mice in the acute toxicity test [198].

## 6. Conclusions

This paper provides a comprehensive review of the current status of alginate and its progress in drug and protein delivery. Alginate as a potential carrier has been investigated for the delivery of a variety of low and high molecular weight drugs. Applications of alginate polymer in pharmaceutical and biomedical research have a promising future. The most important properties of alginate include safety, biocompatibility, and simple methods of preparations. This review highlights the recent advances in the alginate polymers in pharmaceutical and biomedical fields. Because of its biocompatibility, biodegradability, and nontoxicity, it is applied to various drug-delivery technologies. Thus, researchers need to update the advances in the alginate-based drug-delivery systems and this review is a source of guidance for future research.

## Figures and Tables

**Table 1:** Drugs or substances encapsulating in alginate nanoparticles/microparticles.

Drug/protein/substances	Polymer	Aims of encapsulation	References
*Nanoparticles*			
Indomethacin	Alginate-mesoporous silica	Sustained drug-delivery system for poorly water-soluble drug	[53, 54]
Bacteriophages	Alginate-nanohydroxyapatite	Delivery system to prevent orthopedic implant-associated infections	[55]
Bacteriophage	Alginate-CaCO_3_	Encapsulation of bacteriophages	[56]
VEGF	Alginate	Injectable hydrogels for implant	[57]
Prednisolone and inulin	Alginate-chitosan	Nanoparticles for colon delivery	[58]
Amphotericin B	Sodium alginate glycol chitosan stearate	Nanoparticles for better chemotherapy in visceral leishmaniasis	[59]
R6G	Sodium alginate and hydroxyapatite (HAP)	The HAP@Alg nanoparticles show significant potential for the intracellular controlled release of cell-membrane-impermeable drugs	[60]
Dasatinib and zein-lactoferrin	Sodium alginate	Nano-in-micro drug-delivery system for anticancer	[61]
Curcumin and resveratrol	Alginate	Evaluation against DU145 prostate cancer cell line	[62]
Amygdalin	Alginate-chitosan	Biocompatible drug-delivery carriers for anticancer	[63]
5-Fluorouracil	Alginate	Treatment for colon cancer liver metastasis	[64, 65]
Doxorubicin hydrochloride	Alginate/CaCO_3_/DNA	Mediate gene transfection and deliver drug to the cells for cancer treatments	[66]
Tilmicosin	Sodium alginate and carboxymethyl chitosan (CMCS)	The novel TIL-nanogel for treatment of *Staphylococcus aureus* (*S. aureus*) cow mastitis	[67]

*Microparticles*			
Bismuth sulfide	Alginate	Microfluidic alginate microspheres and photothermal effect	[41]
Polystyrene	Sodium alginate	Microspheres of 400 *µ*m to 900 *µ*m produced pH-responsive smart drug-delivery systems	[68]
Gold NPs	Sodium alginate	Alginate hydrogels of higher than 10 nm released PEG-AuNPs for diagnostic and therapeutic purposes	[69]
D-Mannitol	Sodium alginate, sodium cellulose sulfate (SCS), and poly(methylene-co-cyanoguanidine) hydrochloride (PMCG)	Alginate microbeads of 600 to 800 *μ*m stabilized by two coexisting networks for the treatment of diabetes or others	[70]
Sorbitan ester-based organogels	Alginate	Organogels in alginate microparticles	[71]
Corticosteroids	Alginate	Microparticles for colon delivery	[72]
Vancomycin	Chitosan-alginate polyelectrolyte	Vancomycin-chitosan-alginate polyelectrolyte microparticles as the controlled drug-delivery system	[73]

*Other substances*			
Allogeneic pancreatic islet	Alginate	Long-term immune protection of allogeneic pancreatic islet cells	[74]
Lactoferrin	Alginate	Target *Clostridioides difficile* infection	[75]
Probiotic bacteria	Alginate and silica	Freeze-dried microparticles	[76]
Micronutrient	Alginate and chitosan	Functionalization for micronutrient	[77]
*E. coli* Nissle (EcN)	Sodium alginate and chitosan	Alginate-chitosan microcapsule enhanced the survival of EcN	[78]
Cefdinir	Alginate	Floating system and Box–Behnken design	[79]
MICP bacterial spores	Alginate	Self-healing concrete	[80]
SiRNA	Alginate	Vaginal delivery using the scaffold system	[81, 82]
*Bacillus subtilis*	Alginate-chitosan	Alginate microcapsule for uranium ion absorption	[83]
Hyaluronate	Alginate	Regenerating cartilage	[84]

**Table 2:** Alginate nano/microparticles with protein content.

Protein types	Polymer	Method for encapsulation	Significant findings	References
Salmonella effector enzyme (AvrA)	Alginate-chitosan	Microfluidics	Capable of releasing AvrA NPs in the small intestine and colon	[97]
Silk fibroin	Alginate and PLGA	Layer-by-layer deposition	Silk coatings provide stable-encapsulated protein	[98]
Bovine serum albumin	Alginate-poloxamer	Spray drying	Spherical in shape with a size range of 4–6 *μ*m and faster protein release	[99]
Bovine serum albumin	Alginate	Microemulsions-based reactors	Microemulsions of 6 nm stabilized the protein	[100]
Dextran-HEMA	Alginate	Partial oxidation	Good gelling ability	[101]

**Table 3:** Cell studies using alginate nano/microparticles.

Cell types	Polymer	Parameter study	Significant findings	References
Tumor therapeutic cells	Alginate	Encapsulation of cytotoxic compounds encapsulated into liposomes, micelles, and nanoparticles	Long-time release of nanoparticles in the brain parenchyma	[16]
Epithelial cells	Alginate	Physicochemical characteristics and biological properties of the airways	Solubility, lipophilicity, and therapeutic efficacy of microparticles Shape, size, and density have an impact on the microparticles	[19]
Cell-dispersed collagen	Alginate	Microfluidic-based by anisotropic gelation of the capillary	Magnetic-responsive nanoparticles or cell-dispersed collagen for tissue scaffold was functionalized microsprings	[21]
Pancreatic rat islets	Alginates	Cell encapsulation by zwitterionic group	Alginates improved outcome of islet encapsulation in a chemically induced diabetic mouse model	[22]
Riboflavin	Sodium alginate and furfurylamine	Coupling and photo-crosslinked method	Photo-crosslinked F-alginate resulted in slow release and potential for cell growth enhancement for medical application, biomaterials, soft and hard tissue applications, and tissue interfaces	[102]

**Table 4:** Route of administration of drug delivery.

Drugs	Polymer	Route	Formulation/design approach	References
Quercetin	Na alginate and chitosan	Oral	Ionic crosslinking method for oral controlled release	[105]
Isoniazid and rifampicin	Sodium alginate	Oral	Drop technique for oral sustained delivery carriers	[106, 107]
4-(2-Aminoethyl) benzoic acid	Sodium alginate	Oral	Chemically modified (amidation and reductive amination)	[108]
Ciprofloxacin	Alginate-gelatin	Oral	Crosslinked method	[109, 110]
Bovine insulin	Sodium alginate	Oral	Ionotropic gelation using calcium chloride dihydrate	[111]
Lentivectors	Alginate	Oral	Polymers were ionically crosslinked to create bimodal hydrogel	[112]
Resveratrol	Alginate	Oral	Ionic and shelled with soy protein isolate (SPI)	[5]
Metformin	Alginate	Oral	DDS for oral antidiabetic	[113]
Metronidazole	Alginate	Oral	Matrix for oral DDS	[114]
Recombinant hepatitis B surface antigen (rHBsAg)	Alginate	Parenteral	Antigen delivery system for intramuscular administration by mild ionic crosslinking technique	[8]
Furosemide	Alginate-chitosan	Parenteral	Mucopenetrating nanoparticles for enhancement of oral bioavailability	[115]
Exemestane	Sodium alginate	Parenteral	Simple controlled gelation method for oral chemotherapeutic drug	[116]
Paclitaxel	Alginate	Pulmonary	Emulsification technique	[117]
Isoniazid rifampicin, pyrazinamide, and paclitaxel	Chitosan, alginate, PLGA, and polysaccharides	Pulmonary	Emulsification and complexation	[118]
Amikacin, ciprofloxacin, and polymyxin	PLGA and alginate	Pulmonary	Spray drying	[119]
Cisplatin and doxorubicin	Alginate, HAS, chitosan, and PLGA	Pulmonary	Emulsification/gelation and spray drying	[120]
Ciprofloxacin	Polyethylene glycol, phthaloyl chitosan, and sodium alginate	Pulmonary	Grafted and spray drying	[121]
BCG vaccine	Alginate	Pulmonary	Emulsification	[122]
Tobramycin	Alginate and chitosan	Pulmonary	Precipitation	[123]
BCG vaccine	Alginate	Pulmonary	Aerosol liquid encapsulation	[124]
BSA	Alginate	Pulmonary	Spray drying	[99]
BSA	Alginate, chitosan, and trimethyl chitosan	Pulmonary	Liposomal formulation	[125]
Ciprofloxacin	Calcium alginate	Transdermal	Lyophilized hydrogels for wound dressing	[126]
Resveratrol	Chitosan, alginate, and poly(d,l-lactide-co-glycolide)	Transdermal	Nanoprecipitation	[127]
Metronidazole	Alginate	Transdermal	Ionotropic gelation combination with freeze-thawing cycle	[128, 129]

**Table 5:** Various techniques used to produce alginate nanoparticles.

Drugs	Polymer	Method	Size	Main findings	References
Recombinant hepatitis B surface antigen (rHBsAg)	Sodium alginate	Ionic crosslinking	80–400 nm	Size and surface charge could be modulated by adjusting the ratio of polymer	[155]
Curcumin	Alginate, chitosan, and pluronic	Ionic gelation	100 ± 20 nm	Composite nanoparticles (NPs) were successfully prepared	[156]
Doxorubicin	Alginate and chitosan	Novel ionic gelation method	100 nm	Chitosan-alginate nanoparticle produced higher zeta potential and encapsulation efficiency than chitosan nanoparticles	[157]
Hyaluronic acid	Chitosan and alginate	Ionic gelation	100 nm	Cryoprotectants provided stability for the NPs	[158]
Tobramycin	Alginate and chitosan	Isothermal titration calorimetry	±500 nm	High survival rates and low toxicity were observed	[159]
ZnO	Alginate	Pumped dropwise using a peristaltic pump and tubing	120 to 236 nm	Inactivation of antibiotic-resistant bacteria by ZnO NP-alginate beads was improved by increasing the nanocomposite amount and contact time	[160]
Curcumin-loaded zein	Sodium caseinate (SC) and sodium alginate (SA)	Liquid-liquid dispersion and encapsulation	nm	A significantly improved encapsulation efficiency and controlled release was successfully produced	[161]
*trans*-Cinnamaldehyde	Chitosan-alginate	Ionic gelation and polyelectrolyte complexation technique	166.26 nm	(i) Small size and high encapsulation efficiency was found	[162]
Imazapic and imazapyr herbicides	Alginate/chitosan and chitosan/tripolyphosphate nanoparticles	Ionic encapsulation	400 nm	(ii) High efficiency and stable nanoparticles resulted during 30 days of storage at ambient temperature	[163]
Genipin	Silver nanoparticles (AgNPs)-loaded alginate in gelatin scaffolds	Electrospraying and freeze-drying	154 and 171 *μ*m	Swelling and weight loss behaviors of the AgNPs-loaded alginate beads embedded in gelatin scaffolds increased and nontoxic as wound dressings	[164, 165]
Vancomycin (VCM) and glyceryl tripalmitate	Oleic acid (OA), chitosan (CHT), and sodium alginate (ALG)	Hot high-pressure homogenization followed by ultrasonication	202.5 ± 3.81 to 250.9 ± 9.04	(i) Rod-shaped LPNs with suitable size, PDI, zeta potential, higher encapsulation efficiency, and potency as antibacterial activity	[87]
CM-chitin	Polypyrrole (PPY)/sodium alginate	Oxidative polymerization and templating	117–217 ± 17 nm	(ii) Negative viscosity change of the dispersions resulting in a decrease in bulk alginate concentration	[166]
